# Supplementary material for: Predictive olfactory learning in Drosophila
Source: Sci Rep. 2021 Mar 24;11:6795. doi: 10.1038/s41598-021-85841-y (PMC7990964; doi:10.1038/s41598-021-85841-y)
Supplement: Supplementary file 1 — Supplementary Information [file 41598_2021_85841_MOESM1_ESM.pdf]

Supplementary Information

Predictive olfactory learning in *Drosophila*

Chang Zhao[1,+], Yves F. Widmer[2+], Sören Diegelmann[2], Mihai A. Petrovici[1], Simon G. Sprecher[2*], and Walter Senn[1*]

[1] University of Bern, Department of Physiology, Bern, 3012, Switzerland
[2] University of Fribourg, Department of Biology, Fribourg, 1700, Switzerland
[*] corresponding authors: senn@pyl.unibe.ch, simon.sprecher@unifr.ch
[+] these authors contributed equally to this work

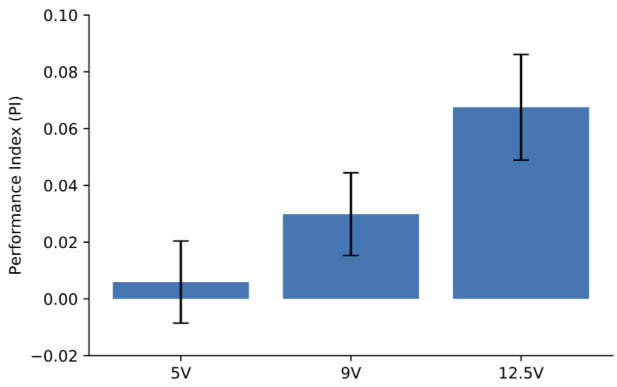

Figure 2-1: The minimal detectable shock stimulus: Electrical shocks with 5V, 9V, and 12.5V are applied in the electric shock avoidance tests. The performance index decreases as the shock intensity decreases. PI is close to 0 when the shock intensity is 5V and significantly above 0 for 9V, and we estimate $S_\circ \approx 7$V. The error bars represent the SEM.

Analytical solution for the ongoing shock experiments

In the ongoing shock experiments, the odor and shock stimuli are both turned on for the whole pairing duration, and turned off when pairing stops. For a constant odor concentration $o = 1$ for $t \geq 0$ while $o = 0$ before, the dynamics of the odor eligibility trace, $\tau_o \dot{\tilde{o}} = -\tilde{o} + o$, is solved by

$$\tilde{o}(t) = o(1 - e^{-\frac{t}{\tau_o}})\,. \tag{S1}$$

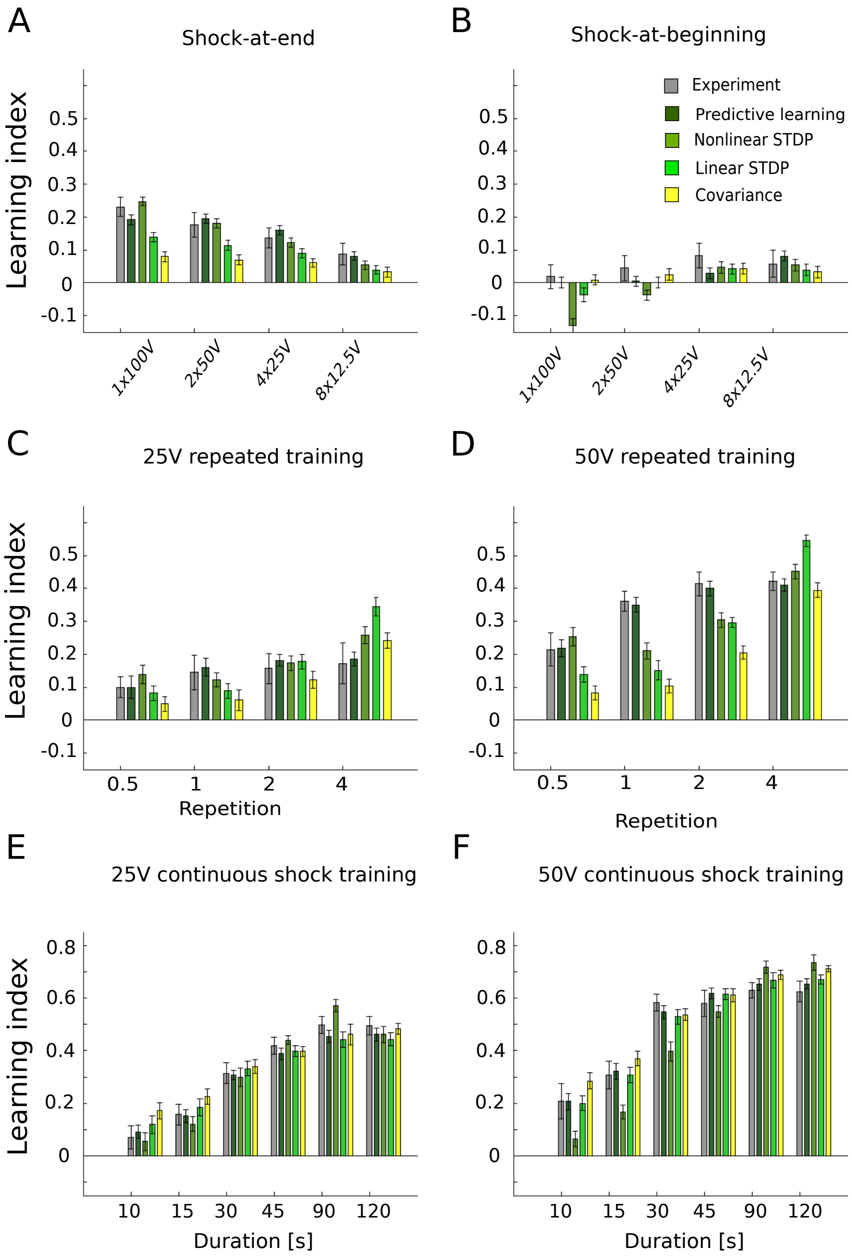

Figure 3-1: Associative learning rules are not able to fit all experimental data. A comparison of all learning rules. **(A)** Temporal sequence training with shocks-at-end alignment. **(B)** Temporal sequence training with shocks-at-beginning alignment. **(C)** Repeated training with 25V. **(D)** Repeated training with 50V. **(E)** Continuous shock training with 25V. **(F)** Continuous shock training with 50V. The associative (linear and nonlinear STDP and covariance) learning rules fail mostly in the repeated training experiments, as they are not able to capture the saturation in the experimental data.

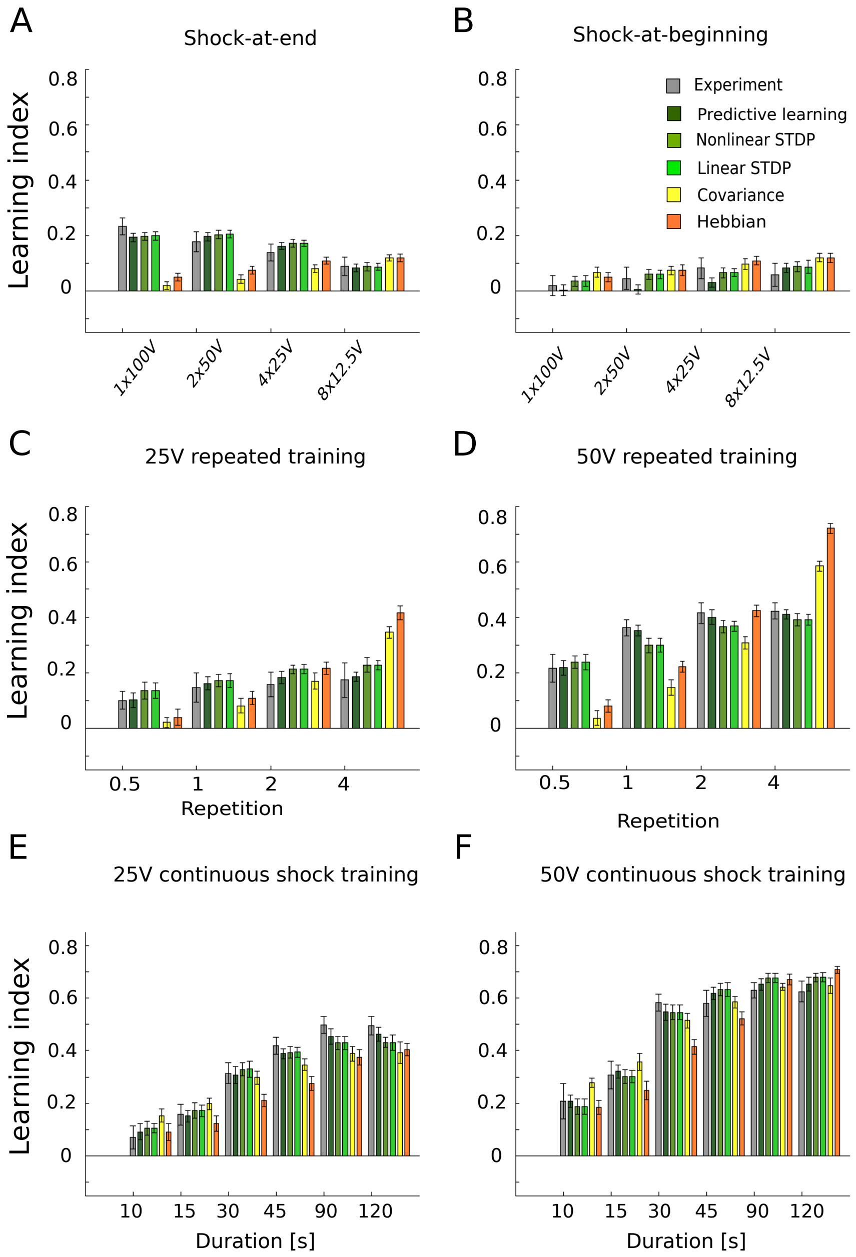

Figure 3-2: Associative learning rules with adaptive learning rate. A comparison of all learning rules. **(A)** Temporal sequence training with shocks-at-end alignment. **(B)** Temporal sequence training with shocks-at-beginning alignment. **(C)** Repeated training with 25V. **(D)** Repeated training with 50V. **(E)** Continuous shock training with 25V. **(F)** Continuous shock training with 50V. The covariance rule and simple Hebbian rule is not able to reproduce all the data. The linear and nonlinear STDP rules perform better with adaptive learning rate, but still have twice as big as MSE comparing to the predictive plasticity rule

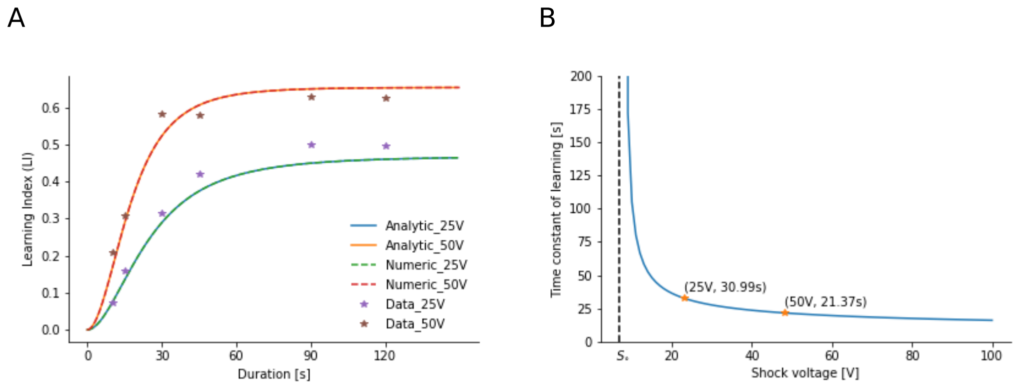

Figure 4-1: Extracting the learning time constant for the ongoing shock experiments. **(A)** The analytical solution (solid lines, Equation S5) for the development of the LI matches the numeric simulation (dash lines, overlaid) for the ongoing conditioning experiments (stars). **(B)** The time constant of learning diverges for shock intensity S close to $S_\circ$, and it monotonically decreases for shock intensities beyond $S_\circ$. For 25V, the learning time constant is 30.99s; for 50V, it is 21.37s (with optimized parameters from the model, see caption of Fig. 3).

With a step increase of the shock from 0 to Δs at time $t = 0$, the learning rate η according to the dynamics Equation 12 undergoes a step increase by $\Delta\eta\Delta s$ that again decays during the constant voltage application,

$$\eta(t) = \Delta\eta\Delta s e^{-\frac{t}{\tau_\eta}} . \tag{S2}$$

The weight w from the KCs to the MBONs Further, according to the predictive plasticity rule Equation 7, $\dot{w} = \eta(s - v)\tilde{o}$, exponentially increases from 0 to $\frac{s}{o}$,

$$w(t) = \frac{s}{o}\left(1 - e^{-f(t)}\right) , \tag{S3}$$

with

$$f(t) = \Delta\eta\Delta s\, o^2 \left(\frac{\tau_\eta \tau_o}{\tau_\eta + \tau_o} \left(e^{-\left(\frac{1}{\tau_\eta} + \frac{1}{\tau_o}\right)t} - 1 \right) - \tau_\eta \left(e^{-\frac{t}{\tau_\eta}} - 1 \right) \right) . \tag{S4}$$

Plugging Equation 19 into $v = wo$ and this into expression for the avoidance probability $p_{cs}(v)$, Equation 5, the learning index $\mathrm{LI}(v) = 2p_{cs}(v) - 1$ develops in time according to

$$\mathrm{LI}(t) = \frac{1 - e^{-s(1-e^{-f(t)})}}{1 + e^{-s(1-e^{-f(t)})}} , \tag{S5}$$

and this converges to $\mathrm{LI}(s)$ is as in Equation 8.
